# A cross-disease meta-GWAS identifies four new susceptibility loci shared between systemic sclerosis and Crohn’s disease

**DOI:** 10.1038/s41598-020-58741-w

**Published:** 2020-02-05

**Authors:** David González-Serna, Eguzkine Ochoa, Elena López-Isac, Antonio Julià, Frauke Degenhardt, Norberto Ortego-Centeno, Timothy R. D. J. Radstake, Andre Franke, Sara Marsal, Maureen D. Mayes, Javier Martín, Ana Márquez, Scleroderma Genetic Consortium, Shervin Assassi, Xiaodong Zhou, Filemon K. Tan, Frank C. Arnett, John D. Reveille, Olga Gorlova, Wei V. Chen, Jun Ying, Peter K. Gregersen, Annette T. Lee, Alexandre E. Voskuyl, Jeska de Vries-Bouwstra, Cesar Magro-Checa, Jasper Broen, Bobby P. C. Koeleman, Carmen P. Simeón, Vicente Fonollosa, Alfredo Guillén, Patricia Carreira, Iván Castellví, Miguel A. González-Gay, Raquel Ríos, Jose Luis Callejas-Rubio, José A. Vargas-Hitos, Rosa García-Portales, María Teresa Camps, Antonio Fernández-Nebro, María F. González-Escribano, Francisco José García-Hernández, Ma. Jesús Castillo, Ma. Ángeles Aguirre, Inmaculada Gómez-Gracia, Luis Rodríguez-Rodríguez, Benjamín Fernández-Gutiérrez, Paloma García de la Peña, Esther Vicente, José Luis Andreu, Mónica Fernández de Castro, Francisco Javier López-Longo, Lina Martínez, Gerard Espinosa, Carlos Tolosa, Anna Pros, Mónica Rodríguez-Carballeira, Francisco Javier Narváez, Manel Rubio-Rivas, Vera Ortiz-Santamaría, Ana Belén Madroñero, Bernardino Díaz, Luis Trapiella, Adrián Sousa, María Victoria Egurbide, Patricia Fanlo-Mateo, Luis Sáez-Comet, Federico Díaz-González, Vanesa Hernández, Emma Beltrán, José Andrés Román-Ivorra, Elena Grau, Juan José Alegre-Sancho, Francisco J. Blanco-García, Natividad Oreiro, Mayka Freire, Alejandro Balsa, Ana M. Ortiz, Nicolas Hunzelmann, Gabriela Riemekasten, Jörg H. W. Distler, Torsten Witte, Paolo Airó, Lorenzo Beretta, Alessandro Santaniello, Chiara Bellocchi, Claudio Lunardi, Gianluca Moroncini, Armando Gabrielli

**Affiliations:** 1 Instituto de Parasitología y Biomedicina López-Neyra, Consejo Superior de Investigaciones Científicas (CSIC), PTS, Granada, Spain; 2 Medical Genetics department, University of Cambridge, Cambridge, UK; 3 Rheumatology Research Group, Vall d’Hebron Research Institute, Barcelona, Spain; 4 Institute of Clinical Molecular Biology, Christian-Albrechts-University of Kiel, Kiel, Germany; 5 Systemic Autoimmune Diseases Unit, Hospital Universitario San Cecilio, Granada; School of Medicine, University of Granada, Instituto de Investigación Biosanitaria ibs.GRANADA, Granada, Spain; 6 Department of Rheumatology and Clinical Immunology, University Medical Center Utrecht, Utrecht, The Netherlands; 7 Division of Rheumatology and Clinical Immunogenetics, The University of Texas Health Science Center-Houston, Houston, TX USA; 8 Systemic Autoimmune Disease Unit, Hospital Universitario San Cecilio, Instituto de Investigación Biosanitaria ibs.GRANADA, Granada, Spain; 9 The University of Texas MD Anderson Cancer Center, Houston, TX USA; 10 The Feinstein Institute for Medical Research, North Shore – Long Island Jewish Health System, Manhasset, NY USA; 11 Department of Rheumatology, VU University Medical Center, Amsterdam, The Netherlands; 12 Department of Rheumatology, Leiden University Medical Center, Leiden, The Netherlands; 13 Radboud University Nijmegen Medical Centre, Nijmegen, the Netherlands; 14 Department of Genetics, Center for Molecular Medicine, University Medical Center Utrecht, Utrecht, the Netherlands; 15 Autoimmune Diseases Unit, Department of Internal Medicine, Hospital Universitario Vall d’Hebron, Barcelona, Spain; 16 Reumatology Department, 12 de Octubre University Hospital, Madrid, Spain; 17 Rheumatology Unit, Santa Creu i Sant Pau University Hospital, Barcelona, Spain; 18 Epidemiology, Genetics and Atherosclerosis Research Group on Systemic Inflammatory Diseases, IDIVAL, University of Cantabria, Santander, Spain; 19 Systemic Autoimmune Diseases Unit, Department of Internal Medicine, Hospital Virgen de las Nieves, Granada, Spain; 20 Rheumatology Department, Hospital Virgen de la Victoria, Málaga, Spain; 21 Internal Medicine Department, Hospital Carlos Haya, Málaga, Spain; 22 Rheumatology Department, Hospital Carlos Haya, Málaga, Spain; 23 Department of Immunology, Hospital Universitario Virgen del Rocío (IBiS, CSIC, US), Sevilla, Spain; 24 Department of Internal Medicine, Hospital Universitario Virgen del Rocío, Sevilla, Spain; 25 Hospital Reina Sofía/IMIBIC, Córdoba, Spain; 26 Instituto de Investigación Sanitaria del Hospital Clínico San Carlos (IdISSC), UGC de Reumatología, Hospital Clínico San Carlos, Madrid, Spain; 27 Rheumatology Department, Madrid Norte Sanchinarro Hospital, Madrid, Spain; 28 Department of Rheumatology, La Princesa Hospital, Madrid, Spain; 29 Department of Rheumatology, Hospital Puerta de Hierro Majadahonda, Madrid, Spain; 30 Department of Rheumatology, Hospital General Universitario Gregorio Marañón, Madrid, Spain; 31 Department of Autoimmune Diseases, Hospital Clínic de Barcelona, Barcelona, Spain; 32 Department of Internal Medicine, Hospital Parc Tauli, Sabadell, Spain; 33 Department of Rheumatology, Hospital Del Mar, Barcelona, Spain; 34 Department of Internal Medicine, Hospital Universitari Mútua Terrasa, Barcelona, Spain; 35 Department of Rheumatology, Hospital Universitari de Bellvitge, Barcelona, Spain; 36 Autoimmune Diseases Unit, Department of Internal Medicine, Hospital Universitari de Bellvitge, Barcelona, Spain; 37 Autoimmune Diseases Unit, Department of Rheumatology, Hospital General de Granollers, Granollers, Spain; 38 Department of Internal Medicine, Hospital General San Jorge, Huesca, Spain; 39 Department of Internal Medicine, Hospital Central de Asturias, Oviedo, Spain; 40 Internal Medicine Department, Hospital Xeral-Complexo Hospitalario Universitario de Vigo, Vigo, Spain; 41 Department of Internal Medicine, Hospital Universitario Cruces, Barakaldo, Spain; 42 Department of Internal Medicine, Hospital Virgen del Camino, Pamplona, Spain; 43 Department of Internal Medicine, Hospital Universitario Miguel Servet, Zaragoza, Spain; 44 Department of Internal Medicine, Hospital Universitario de Canarias, Tenerife, Spain; 45 Department of Rheumatology, Hospital General Universitario de Valencia, Valencia, Spain; 46 Department of Rheumatology, Hospital Universitari i Politecnic La Fe, Valencia, Spain; 47 Department of Rheumatology, Hospital Universitari Doctor Peset, Valencia, Spain; 48 INIBIC-Hospital Universitario A Coruña, La Coruña, Spain; 49 Systemic Autoimmune Diseases and Thrombosis Unit, Department of Internal Medicine, Complexo Hospitalario Universitario de Vigo, Vigo, Spain; 50 Department of Rheumatology, Hospital Universitario La Paz, Instituto de Investigación Sanitaria La Paz. IdiPAZ, Madrid, Spain; 51 Rheumatology Service, Hospital Universitario La Princesa, IIS-IP, Madrid, Spain; 52 Department of Dermatology, University Hospital Cologne, Cologne, Germany; 53 Department of Rheumatology and Clinical Immunology, Charité University Hospital, Berlin, and Department of Rheumatology University of Lübeck, Lübeck, Germany; 54 Department of Internal Medicine, Institute for Clinical Immunology, University of Erlangen-Nuremberg, Erlangen, Germany; 55 University of Hannover, Hannover, Germany; 56 Rheumatology and Clinical Immunology Unit, Spedali Civili, Brescia, Italy; 57 Referral Center for Systemic Autoimmune Diseases, Fondazione IRCCS Ca’ Granda Ospedale Maggiore Policlinico di Milano, Milan, Italy; 58 Department of Medicine, Università degli Studi di Verona, Verona, Italy; 59 Università Politecnica delle Marche and Ospedali Riuniti, Ancona, Italy

**Keywords:** Inflammatory diseases, Risk factors

## Abstract

Genome-wide association studies (GWASs) have identified a number of genetic risk loci associated with systemic sclerosis (SSc) and Crohn’s disease (CD), some of which confer susceptibility to both diseases. In order to identify new risk loci shared between these two immune-mediated disorders, we performed a cross-disease meta-analysis including GWAS data from 5,734 SSc patients, 4,588 CD patients and 14,568 controls of European origin. We identified 4 new loci shared between SSc and CD, *IL12RB2*, *IRF1*/*SLC22A5*, *STAT3* and an intergenic locus at 6p21.31. Pleiotropic variants within these loci showed opposite allelic effects in the two analysed diseases and all of them showed a significant effect on gene expression. In addition, an enrichment in the IL-12 family and type I interferon signaling pathways was observed among the set of SSc-CD common genetic risk loci. In conclusion, through the first cross-disease meta-analysis of SSc and CD, we identified genetic variants with pleiotropic effects on two clinically distinct immune-mediated disorders. The fact that all these pleiotropic SNPs have opposite allelic effects in SSc and CD reveals the complexity of the molecular mechanisms by which polymorphisms affect diseases.

## Introduction

Systemic sclerosis (SSc) and Crohn’s disease (CD) are complex disorders characterized by a chronic deregulation of the immune response, in which both genetic and environmental factors are implicated in their development^1,2^. SSc is a chronic connective tissue disease characterized by vascular injury, excessive collagen deposition and autoantibody production^1^. CD is a chronic autoinflammatory disorder affecting all segments of the gastrointestinal tract, the most common being the terminal ileum and colon^2^.

Even though both diseases present apparently unrelated phenotypic traits, several lines of evidence support the existence of a shared genetic component between them. First of all, results from large-scale genetic studies performed in each individual disease have shown a genetic overlap between SSc and CD, with several genetic risk *loci* common to both conditions, such as *IRF8*, *TYK2*, *STAT4*, and *GSDMA*/*IKZF3*^3,4^. In this regard, the human leukocyte antigen (HLA) region represents one of the most important shared genetic risk loci across immune-mediated diseases^5^, being in fact the major risk *locus* associated with SSc and showing a moderate effect on CD^3,4^. Additionally, there is an important fibrotic component in both diseases. Even when fibrosis is one of the primaries hallmarks of SSc, mainly involving skin, lungs, and gastrointestinal tract, it also appears in CD and is one of the main reasons that leads to a necessity of surgical intervention in the distal part of the small intestine^6,7^. In this line, it has been observed an increased risk of idiopathic pulmonary fibrosis (IPF) in individuals affected by inflammatory bowel diseases, especially in CD patients^8^. Fibrosis of the lungs is one of the most common complications in SSc and, indeed, both IPF and SSc lead to interstitial lung disease (ILD)^9^. Furthermore, the gastrointestinal tract is the internal organ most frequently involved in SSc pathogenesis, which is affected in nearly all patients, sharing this affection with CD. In most of the cases, this affection involves the upper part in SSc and the distal part in CD. However, small bowel and colorectal involvement affects 40–88% and 20–50% of SSc patients, respectively^10,11^, being the distal part of small bowel and colorectum the most affected areas in CD^2^. Thus, these observations suggest that SSc and CD are likely to share common pathogenic mechanisms of disease.

Since the advent of high-throughput genotyping platforms, including genome-wide association studies (GWASs) and the Immunochip approach, more than 15 and 140 genetic risk *loci* have been identified in SSc and CD, respectively^3,4^. However, a significant percentage of the total genetic background of both diseases remains unknown. The low prevalence of immune-mediated disorders represents an obstacle to the identification of their genetic component, making it difficult to recruit well-powered cohorts necessary to detect association signals with weak effects. Cross-phenotype meta-analyses of GWAS or Immunochip data have partially overcome this problem. In recent years, several studies have combined genotypic data from different immune-mediated phenotypes to search for shared risk alleles, either combining paired phenotypes^12–17^ or multiple diseases with common etiology^18–20^. This strategy has allowed the identification of new susceptibility *loci* shared among immune-mediated diseases.

Since no studies analysing the genetic overlap between SSc and CD have been performed so far, the aim of the present study was to thoroughly explore this common genetic background by combining GWAS data from both disorders.

## Methods

### Study population

**Figure 1:**
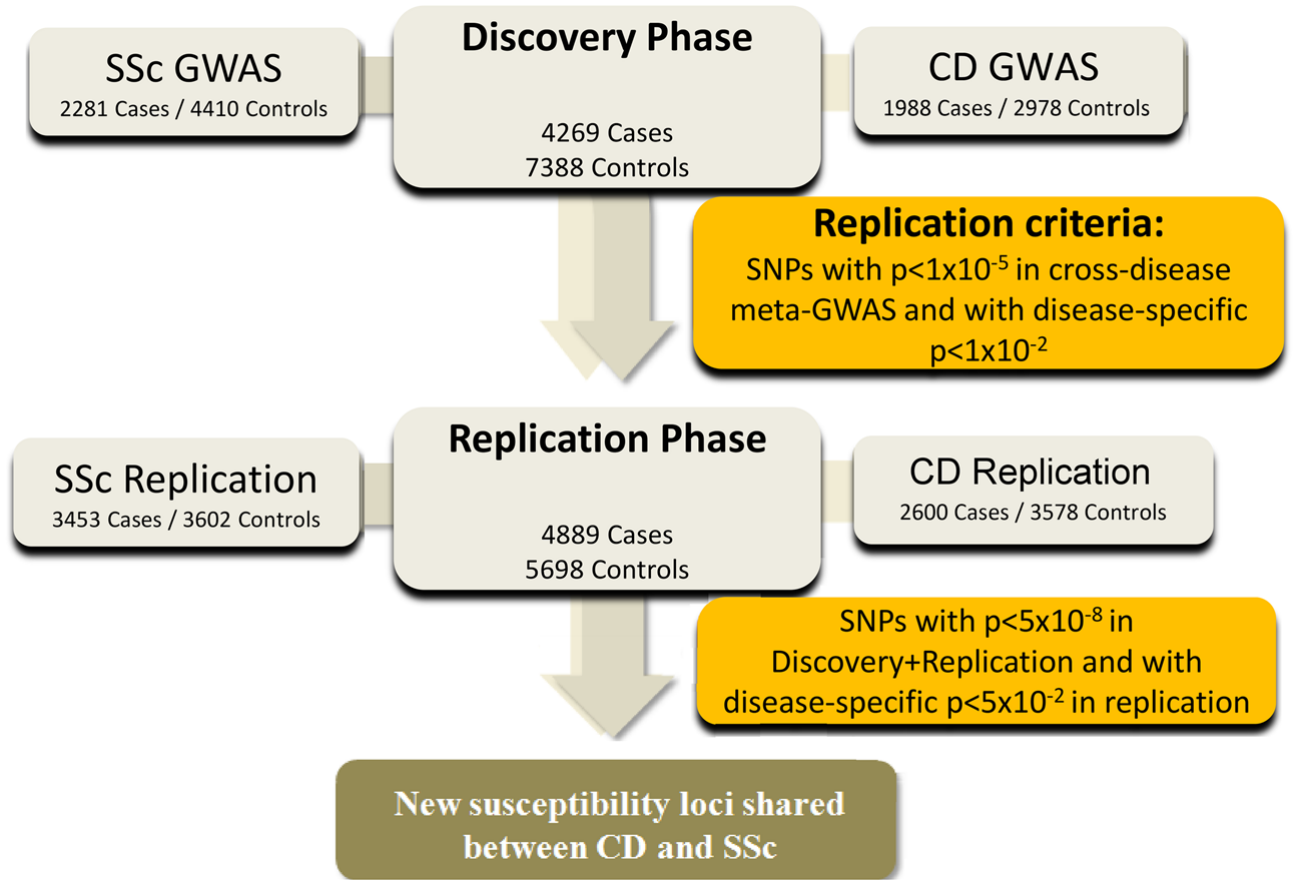
Schema of the study design.

A series of 5,734 patients diagnosed with SSc, 4,588 CD patients, and 14,568 healthy controls of European origin were enrolled in this study. Figure 1 and Supplementary Table S1 detail the cohorts included in the different stages of the study.

#### SSc GWAS dataset

In the discovery phase, we included GWAS data from 2,281 SSc cases and 4,410 healthy controls from Spain, USA, Germany and the Netherlands, all of them included in a previous study^21^ (see Supplementary Table S1).

#### CD GWAS dataset

The CD discovery cohort was composed of 1,988 cases and 2,978 healthy controls from the UK, included in the CD GWAS performed by the Welcome Trust Case Control Consortium (WTCCC)^22^ (see Supplementary Table S1).

#### Replication cohorts

To confirm the results obtained in the discovery phase, genotyping data of the selected polymorphisms were obtained from GWAS data from 3,453 SSc cases and 3,602 controls, and 2,600 CD cases and 3,578 controls. Specifically, the SSc replication cohort included three independent case/control sets from Spain, USA, and Italy. Regarding the CD cohort, case/control sets were recruited from Spain, USA and Germany, all of them from previously published GWASs^23–25^.

The control population consisted of unrelated healthy individuals that were recruited in the same geographical regions as patients. Genotyping information of each cohort is included in Supplementary Table S1.

All SSc cases were defined based on the 1980 preliminary and 2013 classification criteria of American College of Rheumatology^26,27^ or based on the presence of at least 3 out of 5 CREST (calcinosis, Raynaud´s phenomenon, esophageal dysmotility, sclerodactyly, telangiectasias) features typical for SSc. All CD cases were defined based on a confirmed diagnosis of CD using conventional endoscopic, radiological and histopathological criteria^28^.

### Ethics committee approval

Approval from the Comité de Bioética del Consejo Superior de Investigaciones Científicas and the local ethical committees of the different participating centers (University of Texas Health Science Hopkins University Medical Center, Baltimore, USA; Fred Hutchinson Cancer Center-Houston, USA; The Johns Center, Seattle, USA; VU University Medical Center, Amsterdam, The Netherlands; Leiden University Medical Center, Leiden, The Netherlands; Radboud University Nijmegen Medical Centre, Nijmegen, the Netherlands; University Medical Center Utrecht, Utrecht, the Netherlands; Vall d’Hebron Hospital, Barcelona, Spain; 12 de Octubre University Hospital, Madrid, Spain; Santa Creu i Sant Pau University Hospital, Barcelona, Spain; Hospital Marqués de Valdecilla, Santander, Spain; Hospital Clínico Universitario San Cecilio, Granada, Spain; Hospital Virgen de las Nieves, Granada, Spain; Hospital Virgen de la Victoria, Málaga, Spain; Hospital Carlos Haya, Málaga, Spain; Hospital Virgen del Rocío, Sevilla, Spain; Hospital Reina Sofía, Córdoba, Spain; Hospital Clínico San Carlos, Madrid, Spain; Madrid Norte Sanchinarro Hospital, Madrid, Spain; Hospital La Princesa, Madrid, Spain; Hospital Puerta de Hierro Majadahonda, Madrid, Spain; Hospital General Universitario Gregorio Marañón, Madrid, Spain; Hospital Clinic, Barcelona, Spain; Hospital Parc Tauli, Sabadell, Spain; Hospital Del Mar, Barcelona, Spain; Hospital Universitari Mútua Terrasa, Barcelona, Spain; Hospital Universitari de Bellvitge, Barcelona, Spain; Hospital General de Granollers, Granollers, Spain; Hospital General San Jorge, Huesca, Spain; Hospital Central de Asturias, Oviedo, Spain; Hospital Xeral-Complexo Hospitalario Universitario de Vigo, Vigo, Spain; Hospital Universitario Cruces, Barakaldo, Spain; Hospital Virgen del Camino, Pamplona, Spain; Hospital Universitario Miguel Servet, Zaragoza, Spain; Hospital Universitario de Canarias, Tenerife, Spain; Hospital General Universitario de Valencia, Valencia, Spain; Hospital Universitari i Politecnic La Fe, Valencia, Spain; Hospital Universitari Doctor Peset, Valencia, Spain; Hospital Universitario A Coruña, La Coruña, Spain; Hospital Universitario La Paz, Madrid, Spain; Hospital Universitari Germans Trias i Pujol, Badalona, Spain; Hospital General de Alicante, Alicante, Spain; Hospital Clínico Universitario, Zaragoza, Spain; Hospital Clínico Universitario, Santiago de Compostela, Spain; Complejo Hospitalario de León, León, Spain; Hospital de Cabueñes, Gijón, Spain; University Hospital Cologne, Cologne, Germany; Charité University Hospital, Berlin, Germany; University of Erlangen-Nuremberg, Erlangen, Germany; University of Hannover, Hannover, Germany; Spedali Civili, Brescia, Italy; Fondazione IRCCS Ca’ Granda Ospedale Maggiore Policlinico di Milano, Milan, Italy; Università degli Studi di Verona, Verona, Italy; Università Politecnica delle Marche and Ospedali Riuniti, Ancona, Italy; Christian-Albrechts-University, Kiel, Germany) and informed written consent from all participants were obtained in accordance with the tenets of the Declaration of Helsinki. Genome-wide association data from Crohn’s disease patients from UK and USA were obtained from public data repositories, the Wellcome Trust Case Control Consortium (WTCCC) repository and the database of Genotypes and Phenotypes (dbGaP), respectively.

### Quality control and imputation

All GWAS data were quality control (QC) filtered prior imputation. Single-nucleotide polymorphisms (SNPs) and subjects with success call rates lower than 95% were removed using PLINK V.1.9 (www.cog-genomics.org/plink/1.9/)^29^. SNPs showing a deviation from the Hardy–Weinberg equilibrium (*P*-value < 0.001) and minor allele frequencies <1% were also excluded. In addition, one subject per duplicate pair and per pair of first-degree relatives was also removed via the Genome function in PLINK V.1.9 with a Pi-HAT threshold of 0.4. Principal component analysis (PCA) was performed in order to identify and exclude outliers based on their ethnicity by using PLINK V.1.9 and the GCTA64 and R-base under GNU Public license V.2. We estimated the first five PCs using ~100.000 quality-filtered independent SNPs (r^2^ < 0.15). Outliers were defined as individuals who deviated more than six standard deviations from the centroid of their population. The number of SNPs before and after QC for each cohort is summarized in Supplementary Table S1.

Imputation was performed using the Michigan Imputation Server^30^. The software SHAPEIT^31^ was used in order to estimate haplotypes, and the European panel of the Haplotype Reference Consortium r1.1^32^ was used as the reference panel for both SSc and CD genotype data in the discovery phase. Individual chunks of 50.000 Mb were used to carry out the imputation, covering whole-genome regions with a probability threshold for merging genotypes of 0.9, thus maximizing the quality of the imputed variants. Imputed data were also subjected to the above-mentioned QC filters in PLINK V.1.9. The total number of SNPs imputed for each cohort is summarized in Supplementary Table S1.

### Statistical analysis

Statistical analyses were performed with PLINK V.1.9.

#### Discovery phase

Each GWAS case/control cohort was independently analysed by logistic regression assuming an additive model with the first five PCs as covariates, as a correcting method for population stratification. Odds ratios (ORs) and 95% confidence intervals (CIs) were calculated according to Woolf’s method. Subsequently, SSc datasets were meta-analysed by the inverse variance-weighted method. Sex chromosomes were excluded from the analysis.

In order to detect common signals for SSc and CD with the same effect, either risk or protection, we selected SNPs that showed a *P*-value < 1 × 10^−5^ in the SSc-CD meta-analysis and showed nominal significance (*P*-value < 0.01) with each disease separately, as well as no significant heterogeneity in the SSc meta-analysis (Cochran’s Q test > 0.05 and heterogeneity index I^2^ < 50%). To identify common signals for SSc and CD with opposite effect, the direction of association was flipped in the CD dataset (1/OR instead of OR). Again, we selected SNPs that showed a *P*-value < 1 × 10^−5^ in the SSc and CD meta-analysis and that were associated with each disease separately at a *P*-value < 0.01.

The strongest associated SNP within each locus was selected for the replication phase. Genetic variants were annotated using variant effect predictor (VEP)^33^ and their previous association with SSc and/or CD was explored using Immunobase (http://www.immunobase.org) and the GWAS catalog^34^.

#### Replication phase

Replication cohorts were analysed by logistic regression for the previously selected SNPs. Finally, combined analysis of the SSc and CD discovery and replication cohorts was performed using the inverse variance method. After the replication phase, we considered as statistically significant those signals that showed a *P*-value < 0.05 in each disease separately in the replication phase and a *P*-value < 5 × 10^−8^ in the SSc-CD cross-disease meta-analysis including both discovery and replication datasets.

The statistical power of the SSc-CD combined meta-analyses (both discovery and discovery + replication) was determined as described by Skol *et al*.^35^. In the discovery cross-disease meta-analysis, the statistical power to detect an association at a *P*-value of 1 × 10^−5^ (MAF = 20% and OR = 1.2) was 80%. In the discovery + replication meta-analysis, the statistical power to detect an association at a *P*-value of 5 × 10^−8^ (MAF = 20% and OR = 1.2) was 100%.

#### Independence analysis

For those SSc-CD common loci identified for which an association with any of the analysed diseases was already reported, we evaluated the independence between pleiotropic signals and genetic variants previously associated with SSc and/or CD at the genome-wide significance level according to Immunobase and the GWAS Catalog. For this purpose, we used LDlink^36^, a tool that provides linkage disequilibrium (LD) data between polymorphisms across a variety of ancestral populations. Only the European ancestry was taken into account for the LD analysis.

In addition, since one of the shared genetic risk loci was located close to the extended major histocompatibility complex (MHC) region, we decided to test the independence between our new common signal and the main SSc and CD HLA associations. For this, we imputed SNPs, classical HLA alleles and amino acids across the extended MHC region (29,000,000 to 34,000,000 bp in chromosome 6) using the SNP2HLA method with the Beagle software package^37^ and the Type 1 Diabetes Genetics Consortium reference panel, composed of 5,255 individuals of European origin^38^. HLA imputation of the CD discovery cohort was not possible due to the low coverage of this region included in the platform used for the genotyping of this dataset. For the SSc discovery cohort, the presence of independent effects within the extended MHC region was examined using a stepwise logistic regression by conditioning on the top independent signals.

### Functional annotation

We assessed the potential regulatory function of the SSc-CD common susceptibility variants identified by means of in silico expression quantitative trait locus (eQTL) analysis using Haploreg v4.1. Haploreg v4.1 is a tool for exploring annotations at variants on haplotype blocks, providing a large collection of regulatory information, capable of the functional assignment onto any set of variants derived from GWAS or sequencing studies^39^. We only included eQTLs found in tissues with relevance in SSc and/or CD.

### Protein-protein interaction and gene set enrichment analyses

In order to identify interactions among proteins encoded by SSc and CD common risk loci, we decided to construct a protein-protein interaction (PPI) network using the STRING database V.11.0^40^. This software provides a critical assessment and integration of PPI, including functional (indirect) as well as physical (direct) associations.

Gene ontology (GO) was applied to perform an enrichment analysis in order to determine whether certain biological processes are overrepresented in the set of SSc-CD common genes.

## Results

### Meta-analysis and replication

Following QC and imputation, we performed a meta-analysis considering both diseases as a single phenotype. A total of 5,994,231 SNPs overlapped between all GWAS datasets in the discovery phase.

**Figure 2:**
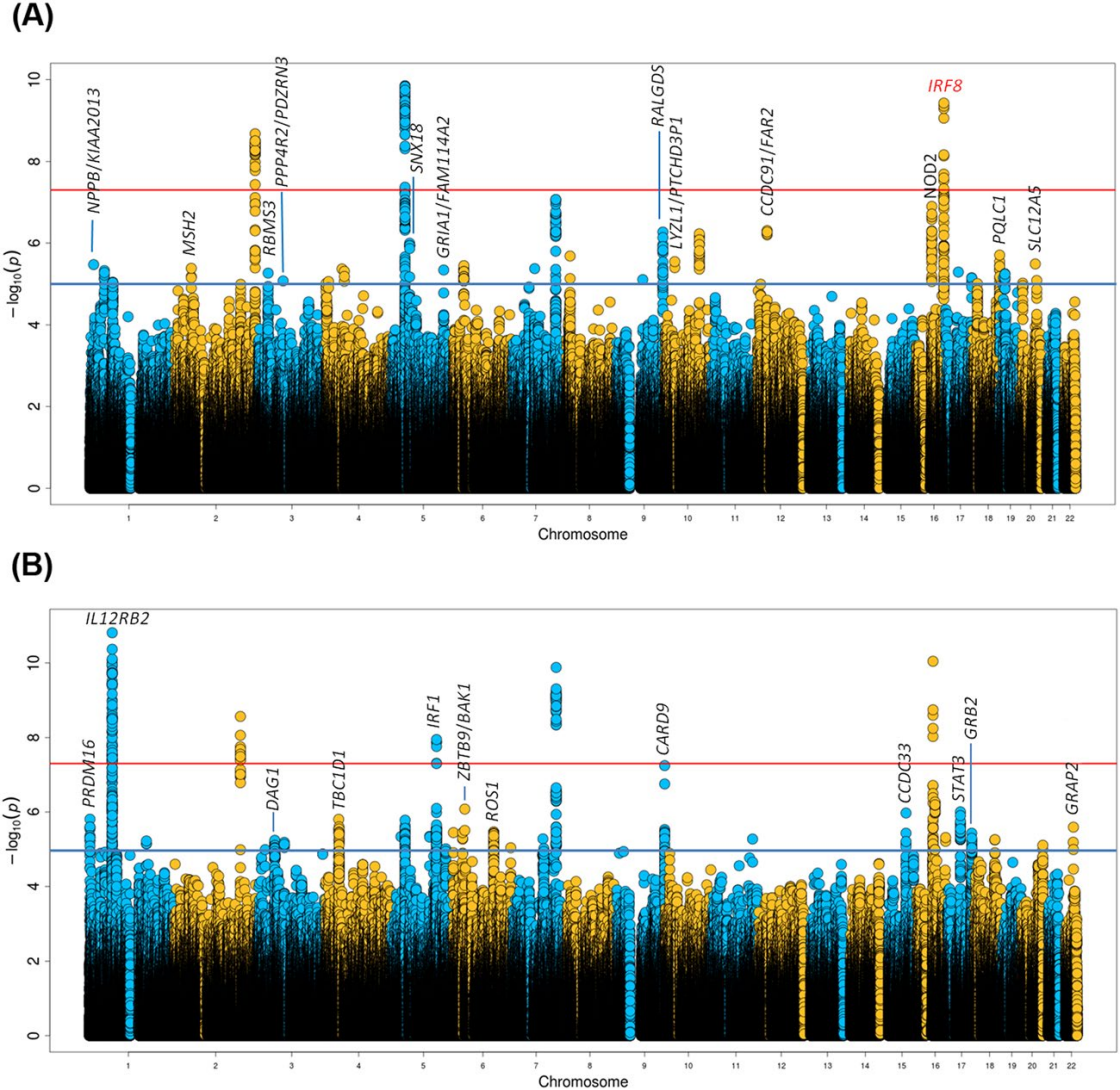
Manhattan plot representing the results of the cross-disease meta-analysis including systemic sclerosis and Crohn’s disease, considering same allelic effects (**A**) and opposite allelic effects (**B**). Loci selected for replication are marked in black. Significance threshold at genome-wide level is marked with a red line. Established significance threshold for the cross-disease meta-analysis (p < 1 × 10^−5^) is marked with a blue line.

When we combined GWAS data from SSc and CD under the assumption that alleles had the same effect in both diseases, genetic variants at 13 *loci* fulfilled the replication criteria (p-value < 1 × 10^−5^ in the SSc-CD meta-GWAS and p-value < 0.01 in each disease-specific analysis) (Fig. 2A and Supplementary Table S2). One of these common signals was located within the *IRF8* region, a known genetic risk locus shared between SSc and CD, and, therefore, it was not considered in subsequent analyses. On the other hand, we performed the analysis under the assumption that alleles had opposite directions in both diseases, identifying 12 *loci* that fulfilled all criteria for the replication phase (Fig. 2B and Supplementary Table S3).

To confirm these associations, the strongest associated SNP within each *locus* was selected for validation in additional sample sets. According to the criteria established for the replication analysis (genome-wide significance in the combined analysis including both discovery and replication sets, and nominal statistical significance in each disease-specific replication analysis), we identified a total of 4 genetic variants showing a pleiotropic effect in SSc and CD: two intronic variants located within *IL12RB2* and *STAT*3, a SNP close to *IRF1*, and an intergenic variant at 6p21.31 located between *ZBTB9* and *BAK1* (Table 1). It is remarkable that an opposite allelic effect in both disorders was observed for all these new common signals.

**Table 1:** Loci associated with a genome-wide significant threshold after the cross-disease meta-analysis of systemic sclerosis and Crohn’s disease.

				Discovery					Replication				Discovery + Replication
				SSc		CD		SSc-CD	SSc		CD		SSc-CD
Region	Gene	SNP	Test Allele	P-value	OR	P-value	OR	P-value	P-value	OR	P-value	OR	P-value
1p31.3	*IL12RB2*	rs6659932	A	2.47E-08	1.3	1.33E-04	0.79	1.54E-11	3.75E-03	1.13	3.44E-02	0.86	1.08E-11
5q31.1	*IRF1*	rs2548998	G	1.55E-03	1.27	3.09E-07	0.79	1.13E-08	2.00E-02	1.08	1.18E-03	0.88	2.18E-11
6p21.31	*ZBTB9/BAK1*	rs68191	C	8.15E-03	0.84	8.70E-06	1.39	8.33E-07	2.09E-04	0.82	1.56E-02	1.15	1.07E-10
17q21.2	*STAT3*	rs4796791	T	1.34E-03	1.13	1.52E-04	0.84	9.86E-07	3.85E-02	1.08	1.85E-02	0.90	2.52E-08

Results of the discovery and replication analysis for each individual disease and of the combined meta-analysis (discovery + replication) are shown.

SNP, single-nucleotide polymorphism; SSc, systemic sclerosis; CD, Crohn’s disease.

Three of these shared risk *loci* have been previously associated with one of the analysed diseases, *IL12RB2* with SSc and *IRF1* and *STAT3* with CD. Shared genetic variants at the *IRF1* and *STAT3* loci identified in our study were linked to those polymorphisms previously associated with CD (r^2^ > 0.40). In the case of *IL12RB2*, it is an established genetic risk *locus* for SSc but, in addition, the *IL23R* gene, located within this same genomic region, is a known susceptibility gene for CD. However, LD analysis evidenced that the pleiotropic variant identified in our study (rs6659932) was independent of the *IL23R* SNPs previously associated with CD (Supplementary Table S4).

On the other hand, the intergenic variant at 6p21.31 (rs68191) is located close to the extended MHC region. Considering this, we decided to test the independence between our new common signal and the main HLA associations observed in the SSc and CD discovery cohorts. In the case of CD, independence between signals could not be checked due to the low coverage of the HLA region. Regarding SSc, two independent signals were observed after conditional regression analysis, HLA-DPB1*1301 (p = 1.77 × 10^−19^, OR = 2.79) and HLA-DRB1*1104 (p = 1.21 × 10^−12^, OR = 1.83). After controlling for these two classical alleles, the SSc-CD common signal remained significant in the SSc discovery cohort (p-value = 8.15 × 10^−3^; conditioned p-value = 2.78 × 10^−2^).

### Functional effect on gene expression

Subsequently, we used the HaploReg database to explor wether the most strogly associated polymorphism of each shared locus acted as an eQTL. As shown in Supplementary Table S5, all the pleiotopic SNPs identified in our study appeared to affect gene expression levels. Shared genetic variants at the *IL12RB2* (rs6659932) and *STAT3* (rs4796791) loci affected expression levels of *IL12RB2* and *STAT3*, respectively, whereas the pleiotropic SNP of the *IRF1* locus (rs2548998) acted as an eQTL for *IRF1* and *SLC22A5*. Interestingly, the intergenic polymorphism at the MHC extended region (rs68191) affected gene expression levels of *TAPBP*.

### Protein-protein interaction and enrichment analysis

Finally, we also evaluated the connectivity at the protein interaction level among the genetic risk loci shared between SSc and CD, including genes whose expression levels were affected by the pleiotopic polymorphisms identified in our study, that is *IRF1*, *SLC22A5*, *STAT3*, *IL12RB2* and *TAPBP*, as well as loci associated in previous studies with both SSc and CD, including *STAT4*, *TYK2*, *IRF8*, *GSDMA* and *IKZF3*. *GSDMA* and *IKZF3* belong to the same LD block, however *GSDMA* has been set as the most probable candidate gene of this locus in SSc and *IKZF3* for CD^41,42^. Thus, we decided to keep both genes for PPI and enrichment analyses.

**Figure 3:**
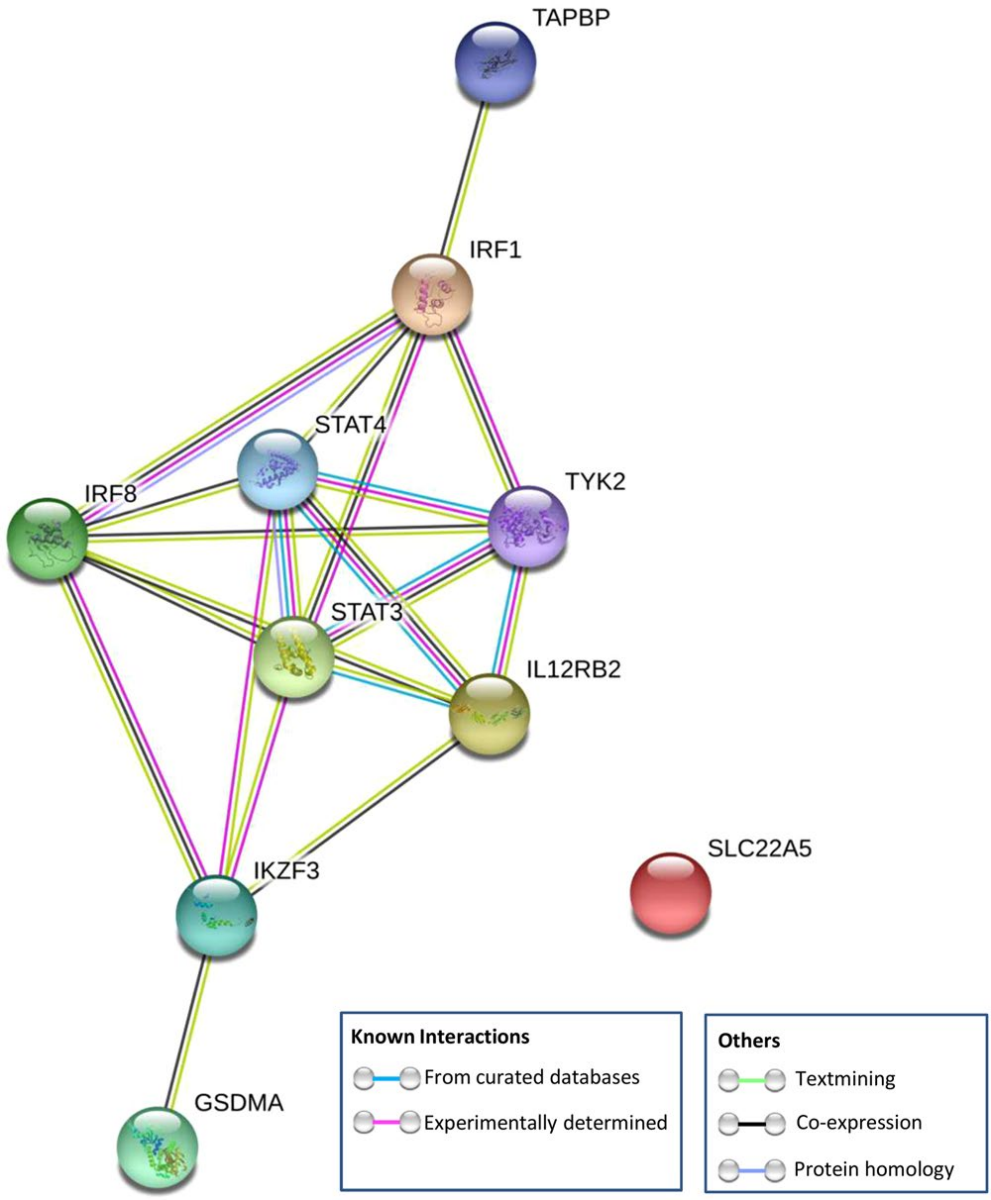
STRING protein-protein interaction network connectivity among genetic risk loci shared between systemic sclerosis and Crohn’s disease.

The PPI network involved 9 of the 10 common proteins included in the analysis, except for SLC22A5 (Fig. 3). We observed a strongly significant PPI enrichment (p-value < 1 × 10^−6^), indicating that these proteins have more interactions than would be expected for a random set of proteins of similar size.

To further evaluate this connection, we performed a gene ontology enrichment analysis in biological processes. In this regard, we observed 29 statistically significant over-represented biological processes (p-value < 0.05). The most significantly over-represented pathways were related to interleukin-mediated signaling, especially those related with the IL-12 family and the type I interferon signaling pathway (Table 2).

**Table 2:** Most significantly enriched Gene Ontology (GO)-biological processes in the set of genetic risk loci shared between systemic sclerosis and Crohn’s disease.

Biological pathway	GO term	p-value*	Count in gene set	Shared genes involved
Interleukin-35-mediated signaling pathway	GO:0070757	1.44E-05	3 of 11	*STAT4*, ***STAT3***, ***IL12RB2***
Interleukin-23-mediated signaling pathway	GO:0038155	1.44E-05	3 of 9	*STAT4*, ***STAT3***, *TYK2*
Cytokine-mediated signaling pathway	GO:0019221	6.16E-05	6 of 655	*STAT4*, ***STAT3***, ***IL12RB2***, *TYK2*, ***IRF1***, *IRF8*
Interleukin-12-mediated signaling pathway	GO:0035722	3.20E-04	3 of 47	*STAT4*, ***IL12RB2***, *TYK2*
Type I interferon signaling pathway	GO:0060337	3.60E-04	3 of 65	*TYK2*, ***IRF1***, *IRF8*
Interleukin-21-mediated signaling pathway	GO:0038114	6.00E-04	2 of 8	*STAT4*, ***STAT3***
Interleukin-27-mediated signaling pathway	GO:0070106	8.30E-04	2 of 11	***STAT3***, *TYK2*
Positive regulation of transcription by RNA polymerase II	GO:0045944	4.90E-03	5 of 1104	*STAT4*, ***STAT3***, ***IRF1***, *IRF8*, *IKZF3*
Positive regulation of interleukin-12 production	GO:0032735	5.90E-03	2 of 34	***IRF1***, *IRF8*
Receptor signaling pathway via JAK-STAT	GO:0007259	7.90E-03	2 of 41	*STAT4*, ***STAT3***
Alpha-beta T cell differentiation	GO:0046632	9.70E-03	2 of 50	***STAT3***, ***IRF1***

*p-values determined by binomial statistic test and adjusted by false discovery rate correction.

New loci shared between systemic sclerosis and Crohn’s disease are in bold.

## Discussion

Through the first comprehensive study of the genetic component shared between SSc and CD, we have identified four *loci* that contribute to suceptibility to both disorders. Of these, one had not been previously associated with any of the diseases under study (an intergenic locus at 6p21.31), whereas the remaining three represent established genetic risk *loci* for one but not the other condition.

Although all these pleiotropic SNPs are located in non-coding regions, functional annotation indicated that they act as regulatory variants affecting expression levels of either the gene were they mapped or close genes in cell types or tissues of relevance in the pathogenesis of SSc and/or CD. In this regard, pleiotropic variants appeared to influence expression levels of the *IL12RB2*, *IRF1*, *SLC22A5*, *STAT3*, and *TAPBP* genes (Supplementary Table S5). Most of these genes are key players of the immune response: *IL12RB2* encodes a subunit of the IL-12 receptor complex implicated in Th1 differentiation; *STAT3* encodes a transcription factor that is essential for the differentiation of Th17 cells; *IRF1* encodes a transcriptional regulator of type I interferon (IFN) and IFN-inducible genes; and *TAPBP* is crucial for optimal peptide loading on the MHC class I molecule. In addition, the pleiotropic variant affecting *IRF1* levels also regulates the expression of *SLC22A5*, which encodes an organic cation transporter involved in the active cellular uptake of carnitine.

Interestingly, PPI analysis evidenced a number of non-random connections among the SSc-CD common genes, including both shared risk loci previously described and comon genes identified in our study, which indicates overlap among the pathways involved in the pathogenesis of these two disorders. Specifically, the IL-12 family signaling pathways, including IL-35, IL-23, IL-12, IL-21, and IL-27-mediated signaling, were particularly compelling. This family of cytokines plays a crucial role in shaping immune responses, differentiation of naïve T cells towards different types of effector cells, as well as in the regulation of effector cell functions^43^. Moreover, the type I interferon signaling pathway was also enriched among the set of SSc-CD common genes. An increased expression and activation of IFN-inducible genes, known as interferon signature, has been reported in SSc^44^ and several interferon regulatory factors (IRFs), including *IRF5*, *IRF4*, and *IRF8*, have been involved in its susceptibility^14,45^, thus supporting the role of *IRF1*, previously associated with CD but not with SSc, as a new susceptibility gene for this last condition.

Considering these results, both IL-12 family and type I interferon signaling pathways could represent interesting therapeutic targets for both SSc and CD. Indeed, ustekinumab, a monoclonal antibody to the p40 subunit common to IL-12 and IL-23, has been recently approved in the EU and the USA to treat patients with CD and, therefore, this drug could be repositioned to treat SSc. However, it should be advised that all the pleiotropic variants identified in our study showed opposite allelic effects in the two analysed disorders, thus highlighting the complex effects that shared associations have on disease outcomes. This could be due to the fact that consequences of genetic variants are influenced by the cell type. For example, as previously indicated, the shared genetic variant at *IL12RB2* influenced *IL12RB2* gene expression levels; however, whereas the minor allele (which conferred risk to SSc in our study) correlated with an increased gene expression in whole blood, the major allele (which conferred risk to CD) had the same effect (increased *IL12RB2* expression) in fibroblasts, according to GTEx data. In addition, the effect on gene expression of the pleiotropic SNP located within the 5q31.1 region was also cell type specific, influencing *IRF1* expression levels in lymphoblastoid cells and *SLC22A5* levels in other tissues, and, therefore, this SNP could have a different biological implication in both diseases. Indeed, higher expression levels of OCTN2, the protein encoded by *SLC22A5*, have been found in inflamed regions of the intestinal epithelium compared with non-inflamed areas, and a role of this protein in the intestinal homeostasis has also been reported^46^; whereas, given the relevance of the type 1 interferon signaling pathway in SSc, the *IRF1* gene seems a more plausible candidate to be involved in SSc susceptibility. Considering this, it is possible that an effective treatment for SSc could have a detrimental effect on CD, and conversely. As previously mentioned, we observed discordant associations for variants located in genes implicated in IL-23 and Th1 differentiation pathways. In this context, IL-17-specific antibody therapy, effective in psoriasis and with promising effects on SSc^47,48^, has been proven to exacerbate CD^49^. This could be due to a deficient Th17 activation in CD owing to mutations in *STAT3*, which could lead to hyper-IgE syndrome, typically associated with extracellular fungal and bacterial infections^50^. Interestingly, according to our results, the *STAT3* rs4796791 variant confers protection to CD and risk to SSc, which could lead to an exacerbate reaction in CD patients carrying this variant when treated with anti-IL17 therapy.

Interestingly, it has been reported a reduced incidence of CD in patients with SSc^51,52^. Although the causes of this phenomenon are not clear, our results suggest that identical genetic risk factors could have different or even opposite functional effects in both diseases. These ‘flip-flop’ associations have been extensively observed across different comparative analyses^53^. In this regard, a cross-disease meta-analysis including CD and type 1 diabetes^54^ identified two variants, such as *IL27* rs4788084 and *IL10* rs3024505, with opposite effects in these two conditions. Furthermore, a meta-analysis of 6 different immune-mediated disorders showed that 14% of overlapped variants were discordant regarding the risk allele across diseases^55^. These results suggest that predisposition to related diseases may be regulated by different dose balance of genes and genomic elements in relevant biological pathways, as well as how these differences affect a specific cell type, as previously mentioned. In this sense, differences across cell types in transcription regulation mediated by epigenetic factors such as methylation, histone modifications or long non-conding RNAs could influence these opposite effects for the same allele in different diseases^56^. It is, therefore, crucial to know the cell types in which genetic variants are acting to be able to elucidate their role on the pathogenesis of the disease.

## Supplementary information

Supplemental material.

## Data Availability

Results of the SSc-CD cross-disease meta-analysis are available from the corresponding author on reasonable request.
