# Supplementary material for: A cross-disease meta-GWAS identifies four new susceptibility loci shared between systemic sclerosis and Crohn’s disease
Source: Sci Rep. 2020 Feb 5;10:1862. doi: 10.1038/s41598-020-58741-w (PMC7002703; doi:10.1038/s41598-020-58741-w)
Supplement: Supplementary file 1 — Supplemental material. [file 41598_2020_58741_MOESM1_ESM.docx]

**A cross-disease meta-GWAS identifies four new susceptibility loci shared between systemic sclerosis and Crohn’s disease**

David González-Serna^1^, Eguzkine Ochoa^1,2^, Elena López-Isac^1^, Antonio Julià^3^, Frauke Degenhardt^4^, Norberto Ortego-Centeno^5^, Scleroderma Genetic Consortium^†^, Timothy RDJ Radstake^6^, Andre Franke^4^, Sara Marsal^3^, Maureen D. Mayes^7^, Javier Martín^1^, Ana Márquez^1,8^.

^1^Instituto de Parasitología y Biomedicina López-Neyra, Consejo Superior de Investigaciones Científicas (CSIC), PTS, Granada, Spain. ^2^Medical Genetics department, University of Cambridge, Cambridge, UK.

^3^Rheumatology Research Group, Vall d'Hebron Research Institute, Barcelona, Spain. ^4^Institute of Clinical Molecular Biology, Christian-Albrechts-University of Kiel, Kiel, Germany. ^5^Systemic Autoimmune Diseases Unit, Hospital Universitario San Cecilio, Granada; School of Medicine, University of Granada, Instituto de Investigación Biosanitaria ibs.GRANADA, Granada, Spain. ^6^Department of Rheumatology and Clinical Immunology, University Medical Center Utrecht, Utrecht, The Netherlands. ^7^Division of Rheumatology and Clinical Immunogenetics, The University of Texas Health Science Center-Houston, Houston, TX, USA. ^8^Systemic Autoimmune Disease Unit, Hospital Universitario San Cecilio, Instituto de Investigación Biosanitaria ibs.GRANADA, Granada, Spain.

**Supplementary Tables**

**Supplementary Table S1**. Study cohorts.

|  |  | **N** | | | | **Genotyping platform** | | | | **Genotyped SNPs** | | **Imputed SNPs** | |
| --- | --- | --- | --- | --- | --- | --- | --- | --- | --- | --- | --- | --- | --- |
|  |  | **SSc** | **Controls** | **CD** | **Controls** | **SSc** | **Controls** | **CD** | **Controls** | **Before QC** | **After QC** | **Before QC** | **AfterQC** |
| **Discovery** | **USA** | 1482 | 2759 | - | - | Illumina HumanHap550K | Breast and prostate cancer controls CGEMS; Illumina iControlDB | - | - | 477,663 | 461,068 | 15,556,150 | 7,347,081 |
|  | **Spain** | 362 | 362 | - | - | Illumina HumanCNV370K | Illumina  HumanCNV370K | - | - | 330,550 | 317,348 | 10,739,039 | 6,885,748 |
|  | **Germany** | 255 | 658 | - | - | Illumina HumanCNV370K | Illumina  HumanHap550k | - | - | 299,278 | 290,928 | 10,236,621 | 7,052,424 |
|  | **Netherlands** | 182 | 631 | - | - | Illumina HumanCNV370K | Illumina  HumanHap550k | - | - | 290,564 | 286,266 | 10,749,243 | 7,143,940 |
|  | **UK** | - | - | 1988 | 2978 | . | - | Affymetrix GeneChip 500K | Affymetrix GeneChip 500K | 380,935 | 379,593 | 11,633,951 | 6,854,680 |
| **Replication** | **USA** | 1286 | 1388 | 956 | 982 | Illumina HumanCore | HumanHap300v1.1 | Illumina HumanHap300k | Illumina HumanHap300k | - | - | - | - |
|  | **Spain** | 1169 | 1262 | 1164 | 1482 | Illumina HumanCore; HumanCytoSNP-12v2 | Illumina HumanCore | Illumina Quad610 Beadchip | Illumina Quad610 Beadchip | - | - | - | - |
|  | **Italy** | 998 | 952 | - | - | Illumina HumanCore | Illumina  HumanHap550k | - | - | - | - | - | - |
|  | **Germany** | - | - | 480 | 1114 | - | - | Illumina HumanHap550k | Illumina HumanHap550k | - | - | - | - |

SSc, systemic sclerosis: CD, Crohn’s disease; QC, quality control.

**Supplementary Table S2**. Genetic variants that reached the replication criteria when considering same allelic effect in systemic sclerosis and Crohn’s disease. Results of each disease-specific meta-analysis and the cross-disease meta-analysis for the strongest associated genetic variant within each locus are shown.

|  |  | **VEP annotation** | |  |  | **SSc** | | **CD** | | **Cross-disease meta-GWAS** | |  |
| --- | --- | --- | --- | --- | --- | --- | --- | --- | --- | --- | --- | --- |
| **Region** | **SNP** | **Consequence** | **Mapped gene** | **Candidate gene** | **Test Allele** | **P-value** | **OR** | **P-value** | **OR** | **P-value** | **OR** | **Previously reported** |
| 16q24.1 | rs11642873 | Intergenic | *-* | *IRF8* | C | 3.78E-07 | 0.77 | 1.99E-04 | 0.81 | 3.73E-10 | 0.79 | SSc, CD |
| 16q12.1 | rs72798422 | Intergenic | *-* | *NOD2* | C | 1.76E-02 | 1.26 | 1.23E-07 | 1.78 | 1.26E-07 | 1.47 | CD |
| 12p11.22 | rs6487699 | Intergenic | *-* | *CCDC91*/*FAR2* | C | 3.45E-05 | 1.17 | 3.92E-03 | 1.14 | 4.92E-07 | 1.16 | - |
| 9q34.2 | rs694881 | Intronic | *RALGDS* | *RALGDS* | G | 2.13E-04 | 0.86 | 7.07E-04 | 0.84 | 5.34E-07 | 0.85 | - |
| 5q11.2 | rs2059214 | Intronic | *SNX18* | *SNX18* | T | 3.73E-05 | 1.19 | 7.07E-03 | 1.14 | 1.00E-06 | 1.17 | - |
| 18q23 | rs72984220 | Intronic | *PQLC1* | *PQLC1* | G | 2.47E-03 | 1.17 | 1.46E-04 | 1.27 | 1.96E-06 | 1.21 | - |
| 10p11.23 | rs11598403 | Intergenic | *-* | *LYZL1*/*PTCHD3P1* | A | 3.37E-03 | 0.83 | 1.39E-04 | 0.75 | 2.83E-06 | 0.8 | - |
| 20q13.12 | rs2297199 | Intronic | *SLC12A5* | *SLC12A5* | C | 3.09E-03 | 1.14 | 7.66E-04 | 1.19 | 3.23E-06 | 1.14 | CD |
| 1p36.22 | rs198382 | Intergenic | *-* | *NPPB/KIAA2013* | C | 5.32E-03 | 1.21 | 9.41E-05 | 1.37 | 3.35E-06 | 1.27 | - |
| 2p16.3 | rs4953504 | Intronic | *MSH2* | *MSH2* | C | 6.78E-03 | 0.87 | 8.87E-05 | 0.79 | 4.13E-06 | 0.84 | - |
| 5q33.2 | rs2614119 | Intergenic | *-* | *GRIA1*/*FAM114A2* | G | 5.10E-03 | 0.9 | 1.55E-04 | 0.85 | 4.54E-06 | 0.88 | - |
| 3p24.1 | rs1347772 | Intronic | *RBMS3* | *RBMS3* | T | 1.98E-04 | 0.87 | 8.22E-03 | 0.88 | 5.41E-06 | 0.87 | - |
| 3p13 | rs6765560 | Intergenic | *-* | *PPP4R2/PDZRN3* | C | 9.78E-04 | 1.13 | 2.66E-03 | 1.14 | 8.42E-06 | 1.14 | - |

VEP, variant effect predictor; SSc, systemic sclerosis; CD, Crohn’s disease.

**Supplementary Table S3**. Genetic variants that reached the replication criteria when considering opposite allelic effect in systemic sclerosis and Crohn’s disease. Results of each disease-specific meta-analysis and the cross-disease meta-analysis for the strongest associated genetic variant within each locus are shown.

|  |  | **VEP annotation** | |  |  |  | **SSc** | | **CD** | | **Cross-disease meta-GWAS** | |  |
| --- | --- | --- | --- | --- | --- | --- | --- | --- | --- | --- | --- | --- | --- |
| **Region** | **SNP** | **Consequence** | **Mapped gene** | **Candidate gene** | **Test Allele** | **Position** | **P-value** | **OR** | **P-value** | **Inverted OR** | **P-value** | **OR** | **Previously reported** |
| 1p31.3 | rs6659932 | Intronic | *IL12RB2* | *IL12RB2* | A | 67802371 | 2.47E-08 | 1.3 | 1.33E-04 | 1.26 | 1.54E-11 | 1.28 | SSc |
| 5q31.1 | rs2548998 | Intronic | *AC116366.3* | *IRF1* | G | 131832514 | 1.55E-03 | 1.27 | 3.09E-07 | 1.26 | 1.13E-08 | 1.18 | CD |
| 9q34.3 | rs3812565 | Upstream | *CARD9* | *CARD9* | C | 139272502 | 1.86E-03 | 0.88 | 2.70E-06 | 0.81 | 5.67E-08 | 0.85 | CD |
| 6p21.31 | rs68191 | Intergenic | - | *ZBTB9/BAK1* | C | 33480738 | 8.15E-03 | 0.84 | 8.70E-06 | 0.72 | 8.33E-07 | 0.79 | - |
| 17q21.2 | rs4796791 | Intronic | *STAT3* | *STAT3* | T | 40530763 | 1.34E-03 | 1.13 | 1.52E-04 | 1.19 | 9.86E-07 | 1.16 | CD |
| 15q24.1 | rs12905224 | Intronic | *CCDC33* | *CCDC33* | C | 74533436 | 2.15E-04 | 1.16 | 1.47E-03 | 1.16 | 1.06E-06 | 1.15 | - |
| 1p36.32 | rs2742661 | Intronic | *PRDM16* | *PRDM16* | T | 3069937 | 6.29E-04 | 1.2 | 6.53E-04 | 1.25 | 1.56E-06 | 1.22 | - |
| 4p14 | rs17578878 | Intronic | *TBC1D1* | *TBC1D1* | T | 37900725 | 9.23E-05 | 1.26 | 5.02E-03 | 1.23 | 1.57E-06 | 1.25 | - |
| 22q13.1 | rs138014 | Downstream | *GRAP2* | *GRAP2* | T | 40373297 | 3.11E-04 | 0.87 | 2.53E-03 | 0.87 | 2.54E-06 | 0.87 | - |
| 6q22.1 | rs72969416 | Intronic | *ROS1* | *ROS1* | C | 117738685 | 2.67E-04 | 0.8 | 3.91E-03 | 0.82 | 3.43E-06 | 0.81 | - |
| 17q25.1 | rs4350602 | Intronic | *GRB2* | *GRB2* | C | 73355769 | 4.91E-04 | 0.86 | 2.27E-03 | 0.85 | 3.69E-06 | 0.86 | - |
| 3p21.31 | rs4625 | Downstream | *DAG1* | *DAG1* | G | 49572140 | 6.82E-03 | 0.9 | 1.36E-04 | 0.83 | 5.72E-06 | 0.87 | CD |

VEP, variant effect predictor; SSc, systemic sclerosis; CD, Crohn’s disease.

**Supplementary Table S4.** Linkage disequilibrium between the systemic sclerosis-Crohn’s disease common signal at *IL12RB2* (rs6659932) and the *IL23R* polymorphisms previously associated with Crohn’s disease.

|  |  | **LD with rs6659932** | |  |
| --- | --- | --- | --- | --- |
| **SNP** | **Position** | **r^2^** | **D'** | **Study accession number** |
| rs7517847 | 1:67215986 | 0.0051 | 0.1372 | GCST003044 |
| rs11581607 | 1:67242007 | 0.0059 | 0.1328 | GCST004132 |
| rs11465804 | 1:67236843 | 0.0124 | 0.1854 | GCST000207 |
| rs11209026 | 1:67240275 | 0.0059 | 0.1328 | GCST001396 |
| rs11805303 | 1:67209833 | 0.0043 | 0.2282 | GCST000042 |
| rs76418789 | 1:67182913 | 0.0004 | 1 | GCST002094 |

**Supplementary Table S5.** Potential role of the lead pleiotropic polymorphisms as expression quantitative trait loci (eQTLs) in tissues of relevance for the diseases under study.

| **Region** | **Locus** | **SNP** | **Correlated gene** | **Tissue** | **p-value** | **Study** |
| --- | --- | --- | --- | --- | --- | --- |
| 5q31.1 | *IRF1* | rs2548998 | *SLC22A5* | Whole Blood | 2.95E-07 | (1) |
|  |  |  |  | Esophagus - Mucosa | 5.09E-06 | (1) |
|  |  |  |  | Cells - Transformed fibroblasts | 6.82E-06 | (1) |
|  |  |  |  | Lymphoblastoid cells | 4.94E-07 | (2) |
|  |  |  | *IRF1* | Lymphoblastoid cells | 1.33E-06 | (2) |
| 1p31.3 | *IL12RB2* | rs6659932 | *IL12RB2* | Cells - Transformed fibroblasts | 3.41E-13 | (1) |
|  |  |  |  | Whole Blood | 5.47E-07 | (1) |
| 17q21.2 | *STAT3* | rs1026916* | *STAT3* | Whole Blood | 1.99E-20 | (3) |
| 6p21.31 | *ZBTB9/BAK1* | rs68191 | *TAPBP* | Whole Blood | 4.04E-06 | (3) |

SNP, single nucleotide polymorphism; *Proxy SNP of the strongest associated polymorphism, rs47967941 (r^2^=0.99).

(1) GTEx Consortium. Science. 2015;348(6235):648-60.

(2) Lappalainen T, et al. Nature. 2013;501(7468):506-11

(3) Westra HJ, et al. Nat Genet. 2013;45(10):1238-43.
